# Clinical practice guideline management of blood borne viruses within the haemodialysis unit

**DOI:** 10.1186/s12882-019-1529-1

**Published:** 2019-10-28

**Authors:** Elizabeth Garthwaite, Veena Reddy, Sam Douthwaite, Simon Lines, Kay Tyerman, James Eccles

**Affiliations:** 1 Leeds Teaching Hospitals NHS Trust, Leeds, UK; 2 Sheffield Teaching Hospitals NHS Foundation Trust, Sheffield, UK; 3 Guy’s and St. Thomas’ NHS Trust, London, UK; 4 Norwich and Norfolk University Hospitals NHS Foundation Trust, Norwich, UK; 5 Patient Representative, c/o The Renal Association, Bristol, UK

## Abstract

Some people who are receiving dialysis treatment have virus infection such as hepatitis B, hepatitis C and/or HIV that is present in their blood. These infections can be transmitted to other patients if blood is contaminated by the blood of another with a viral infection. Haemodialysis is performed by passing blood from a patient through a dialysis machine, and multiple patients receive dialysis within a dialysis unit. Therefore, there is a risk that these viruses may be transmitted around the dialysis session. This documents sets out recommendations for minimising this risk.

There are sections describing how machines and equipment should be cleaned between patients. There are also recommendations for dialysing patients with hepatitis B away from patients who do not have hepatitis B. Patients should be immunised against hepatitis B, ideally before starting dialysis if this is possible. There are guidelines on how and when to do this, for checking whether immunisation is effective, and for administering booster doses of vaccine. Finally there is a section on the measures that should be taken if a patient receiving dialysis is identified as having a new infection of hepatitis B, hepatitis C or HIV.

## Introduction

Blood borne virus (BBV) infection was recognised as an important hazard for patients and staff in renal units in the 1960s [1]. In 1972 the Rosenheim Report was commissioned by the precursor to what is now the Department of Health (DoH) and included a set of guidelines for the control of hepatitis B virus (HBV) infection in renal units [2].

In 2002 a working party convened by the Public Health Laboratory Service (PHLS) on behalf of the Department of Health published an updated report that also included recommendations related to hepatitis C virus (HCV) and human immunodeficiency virus (HIV) infection [3].

The Renal Association Clinical Guidelines on the management of blood borne viruses within the renal unit were published in 2008. These have been revised and updated based on a small body of clinical evidence identified by on-line literature searching of PubMed from 1966 to 2018. Search terms used included haemodialysis, hemodialysis, hepatitis, HIV, transmission, immunisation, vaccination and ‘chronic kidney disease’.

The incidence of HBV and HCV in dialysis units has fallen over the last 3 decades although data from USA showed that the incidence of HBV infection in dialysis units had stayed stable at 1% per year in the 10 years before 2002 [4].

Most UK renal health care workers have probably never witnessed an outbreak of BBV in the renal unit. However, the ever increasing prevalence of patients on haemodialysis [5], the increase in migration of patients from other countries and the relative ease of foreign travel for dialysis patients means that renal units need to be increasingly alert to the possibility of BBV transmission.

A substantial part of the reduction in the incidence of BBV infection in renal units has been associated with the implementation of so-called “universal”, or “standard”, precautions for prevention of BBV transmission. However, there continues to be numerous reports of outbreaks of BBV infection in renal units worldwide and often there is evidence that these have been caused by lapses in high standards of infection control practice [6–11]. There is also anecdotal evidence of cases of hepatitis B ‘reactivation’ when patients with evidence of previous exposure to hepatitis B and native immunity (hepatitis B core antibody positive) reactivate the infection in the context of significant immunosuppression.

The main risks relate to HBV, HCV and HIV infections. These viruses have been associated with outbreaks among patients and staff in haemodialysis units. Other BBV such as Hepatitis G and D have been identified as being more commonly carried in dialysis patients than the general population but their clinical significance is uncertain [12–14].

Risk of BBV transmission is known to be directly related to the concentration of virus in the blood. HCV and HIV are less infectious in dialysis units than HBV but outbreaks have been reported [7, 8, 13–18] emphasising the need for infection control measures. Within the guideline we refer to the KDIGO guidelines for the management of HCV within the renal unit and refer to the specific recommendations for infection control [19].

Patients with any acute BBV infection are probably more infectious than chronic carriers and this guideline therefore includes recommendations to try to identify patients at risk of acute BBV infection.

Most of the evidence to support the recommendations comes from observational clinical studies, case series and in vitro observations. This is largely because the incidence of BBV is low, despite the risks of potential BBV exposure remaining high. When recommending areas for future research we have chosen not to recommend interventional controlled trials that are unfeasible.

From large multicentre and single centre observational studies there is a clear demonstration of the reduction of the incidence of BBV infection in association with the introduction of a range of infection control measures [20–22]. Indeed, the majority of outbreaks in Europe since 2005 have been associated with a breach in infection prevention measures [23–27].

Infection prevention measures demand intensive and careful staffing and are dependent on maintaining our expert workforce. However this is being challenged by constraints on staffing including reduced nurse to patient ratios, and a focus on efficiency saving. The recommendations do take into account the resources that can realistically be expected in UK renal units: e.g. a dialysis nurse to patient ratio of 1:1 would probably reduce the risk of BBV transmission but is not recommended as it is not feasible. However, any proposed changes in staffing ratios in a unit should be accompanied by a risk assessment of the implications of this on the ability to adhere to the infection control measures recommended within this guideline. When applying this clinical practice guideline it is important to consider the balance between protecting patients from the risks of BBV transmission and compromising clinical care of patients infected, or at high risk of infection with BBV especially with regards to segregation.

Within the guideline we have added additional detail regarding the vaccination of patients against HBV infection. At the time of writing there is a UK shortage of hepatitis B vaccine - however the guidelines assume a robust supply of the vaccine and provides recommendations on vaccination procedures and monitoring. There is a clear statement within the guideline that the efficacy of the vaccine is significantly improved when delivered within the pre dialysis setting - though the implementation of this is beyond the scope of the guideline.

This guideline does not cover treatment of BBV in patients with chronic kidney disease (CKD) or prevention of BBV infection in patients receiving kidney transplants.

These guidelines also apply to children less than 16 yrs. of age even though there is a paucity of published data relating specifically to the management of BBV within the paediatric haemodialysis unit/setting. (1D).

## Scope

1. Prevention of BBV infection in the renal unit
  1.1. Infection control procedures
  1.2. Parenteral medicines (single use)
2. Dialysis Equipment and BBV infection
  2.1. Machine segregation for patients infected with HBV
  2.2. Precautions for patients with HCV/HIV
  2.3. Utilisation of external transducers
  2.4. Disinfection process for dialysis equipment
3. BBV surveillance in dialysis patients
  3.1. Virology status of patients starting HD
  3.2. Management of patients starting HD with unknown virology status
  3.3. Surveillance for HBV/HCV/HIV in prevalent HD population
  3.4. Management of patients who do not consent for BBV testing
  3.5. Management of patients returning from dialysis outside UK
  3.6. Procedures for enhanced surveillance of high risk patients
  3.7. Management and surveillance of patients vaccinated against HBV
4. Segregation of patients infected/at risk of infection
  4.1. Isolation of patients known to be infected with HBV
  4.2. Management of patients known to be infected with HCV/HIV
5. Immunisation of patients against hepatitis B
  5.1. Indications for vaccination
  5.2. Immunisation schedule
  5.3. Identification and management of responders/non responders
6. Immunisation of staff against hepatitis B infection
7. Management of a new case of BBV infection on the dialysis unit
  7.1. Management of a new case of HBV infection
  7.2. Management of a new case of HCV infection

## Summary of clinical practice guidelines

### Prevention of BBV infection in the renal unit (guidelines 1.1–1.2)

#### Guideline 1.1- BBV prevention: infection control procedures

The single most important method of prevention of transmission of blood borne viruses is the rigorous application of universal infection control precautions. We recommend that infection control procedures must include hygienic precautions that effectively prevent the transfer of blood or fluids contaminated with blood between patients either directly or via contaminated equipment or surfaces (KDIGO Hepatitis C guideline 3.1) (1A).

#### Guideline 1.2 – BBV prevention: use of parenteral medicines

We recommend that medicine vials should be discarded after single use and multi-use vials should be avoided. If medicine vials are used for more than one patient, we recommend they are divided into multiple doses and distributed from a central area. Intravenous medication vials labelled for single use should not be punctured more than once, as the sterility of the product cannot be guaranteed once a needle has entered a vial labelled for single use (1B).

### Dialysis equipment and BBV infection (guidelines 2.1–2.5)

#### Guideline 2.1 – BBV infection: machine segregation for patients infected with HBV

We recommend that separate machines must be used for patients known to be infected with HBV (or at high risk of new HBV infection). A machine that has been used for patients infected with HBV can be used again for non-infected patients only after it has been decontaminated using a regime deemed effective against HBV. Healthcare workers dialysing patients with known HBV infection should not dialyse patients without HBV infection at the same time (1A).

#### Guideline 2.2 – BBV infection: precautions for patients with HCV/HIV

We recommend that dedicated machines are not required for patients infected with HCV and HIV, provided cleaning and disinfection procedures are strictly adhered to between patients (KDIGO Hepatitis C guideline 3.1.2) (European Renal Best Practice Guidelines) (1D).

#### Guideline 2.3 – BBV infection: utilisation of external transducers

We suggest that external transducer protectors on the blood circuit pressure monitoring lines should be inspected by healthcare personnel during and after each dialysis session. If there is evidence of breach by blood or saline then the machine should be taken out of service and machine components that may have come in contact with blood should be replaced or decontaminated by qualified personnel according to a protocol that incorporates the manufacturers’ instructions (2C).

#### Guideline 2.4 – BBV infection: disinfection process for dialysis equipment

We recommend that the dialysis machine should be cleaned between patients according to a local protocol that incorporates the manufacturer’s instructions (1C).

### BBV surveillance in dialysis patients (guidelines 3.1–3.7)

#### Guideline 3.1 – BBV infection: virology status of patients starting Haemodialysis

We recommend that all patients starting haemodialysis (including patients with acute kidney injury) or returning to haemodialysis after another modality of renal replacement therapy should be known to be plasma HBV surface antigen (HBsAg) negative before having dialysis on the main dialysis unit (1A).

We recommend HCV screening all patients starting haemodialysis or returning to haemodialysis after another modality of renal replacement therapy. We recommend patients with no identified risk factors for acquiring HCV may be screened by an immunoassay. If the immunoassay is positive, we recommend a follow up screen with nucleic acid testing (NAT). Patients with current or historical risk factors for HCV acquisition should initially be screened by NAT, with subsequent reversion to serological methods if no ongoing risk factors are present. NAT screening should be continued in patients with ongoing risk factors (KDIGO Hepatitis C guideline 1.1.2) (1A).

We recommend that HIV screening should be undertaken in all patients starting haemodialysis (1C).

#### Guideline 3.2 – BBV infection: management of patients starting Haemodialysis with unknown virology status

We recommend that patients who require haemodialysis before the result of the HBsAg test is known should be dialysed in an area that is segregated within the main dialysis unit (such as a side room) and the machine should not be used for another patient until the result is known to be negative or the machine has been decontaminated using a HBV suitable decontamination regime (see 2.1) (1A).

#### Guideline 3.3 – BBV infection: surveillance for HBV/HCV/HIV in prevalent Haemodialysis population

We recommend that patients on regular hospital haemodialysis who are immune to hepatitis B immunisation (anti HBs antibody titre > 100 mIU/ml; see section 5 below), only need to be tested for HBsAg every 6 months. Non-responders should be tested at least every 3 months (1C). For ease units may prefer to routinely test for HBsAg every 3 months for all patients.

We recommend that patients on regular hospital haemodialysis should be tested for HCV antibody every 3 months. However, those with historical or current risk factors for HCV acquisition should be tested using a NAT test (1C).

We recommend that antibody surveillance testing for HIV is not necessary for patients on regular hospital haemodialysis unless the patient is at high risk (See Table 4) (1C).

#### Guideline 3.4 – BBV infection: management of patients who do not consent for BBV testing

We suggest that patients who do not consent to BBV surveillance, as described above, should have dialysis in a segregated area unless they are known to be HBV immune in the previous 6 months. If patients who are known to be HBV immune within the previous 6 months do not consent to BBV surveillance then they should be managed in the same way as patients with HCV infection (see section 4) (2C).

#### Guideline 3.5 – BBV infection: management of patients returning from dialysis outside UK

We recommend that patients planning to dialyse outside the UK should have a risk assessment prior to travel for potential exposure to BBV abroad. Where exposure is considered likely, enhanced surveillance testing for BBV should be planned and instituted and patients should have dialysis in a segregated area as detailed below (1B).

#### Guideline 3.6 – BBV infection: procedures for enhanced surveillance of high risk patients

We recommend that patients at high risk for new BBV infection (see Table 4) should have enhanced surveillance as described in section 3.5 (1B).

We recommend that testing for HBsAg and HCV RNA should be performed in haemodialysis patients with unexplained abnormal serum aminotransferase concentrations (KDIGO Hepatitis C guideline 1.2.2) (1B).

We recommend that if a new BBV infection is identified in a haemodialysis unit, testing for viral RNA or DNA should be performed in all patients who may have been exposed (see section 7) (KDIGO Hepatitis C guideline 1.2.4). (1B).

### Segregation of patients infected or at risk of infection with BBV (guidelines 4.1–4.2)

#### Guideline 4.1 – BBV infection: isolation of patients known to be infected with Hepatitis B virus (HBV)

We recommend that patients infected with HBV must be dialysed in an area that is segregated from the main dialysis unit. (1A).

We recommend that healthcare workers performing dialysis on patients infected with HBV infection should not dialyse patients without HBV infection at the same time. (1C). If this is not possible then they must wear disposable PPE and ensure scrupulous attention to hand hygiene before moving from one patient to the other.

#### Guideline 4.2 – BBV infection: management of patients infected with Hepatitis C virus (HCV) or HIV

We recommend that patients with HCV or HIV do not need to be dialysed in a segregated area, providing infection control and universal precautions can be properly adhered to. (1C)KDIGO Hepatitis C guideline 3.1).

### Immunisation of patients against Hepatitis B virus (guidelines 5.1–5.7)

#### Guideline 5.1 – BBV infection: indications for immunisation of patients against hepatitis B virus (HBV)

We recommend that all patients who require renal replacement therapy (RRT) [dialysis or transplantation] for CKD should be assessed for current or past infection with Hepatitis B and offered vaccination against HBV if indicated. (1A).

#### Guideline 5.2 – BBV infection: timing of initiating immunisation schedule against HBV

We recommend that patients who are likely to require RRT, who are deemed susceptible to HBV infection, should be offered vaccination prior to the development of Stage V CKD [or 2 years before they are likely to need renal replacement therapy, as judged by the clinical team managing the patient]. (1A) A kidney failure risk calculator could be used to facilitate this prediction.

#### Guideline 5.3 – BBV infection: identification of patients for whom immunisation against HBV is not indicated

Hepatitis B vaccine is not indicated in patients who have current (Hepatitis B surface antigen (HBsAg) positive or HBV DNA positive) or confirmed past HBV infection. Presence of the anti HBc antibody in isolation should not be taken as confirmation of previous HBV infection. Patients identified to be core antibody positive who are at risk of reactivation of HBV (particularly immunosuppression) may need to be vaccinated and the case should be discussed with a local virologist. (2B).

#### Guideline 5.4 – BBV infection: immunisation schedule for vaccination against Hepatitis B virus

We recommend that the initial HBV immunisation schedule should involve high doses, frequent doses or both of the available preparation (1A).

We recommend that the vaccines are administered intramuscularly as per their licensed route (deltoid muscle) but, if sufficient expertise exists, the intradermal route may more effective. (1A) (Table 1).

**Table 1:** Available vaccines, doses and immunisations schedules (1A)

Vaccine Product	Ages	Dose	Schedule (months)
^a^Engerix B®	0-15 yrs	10micrograms	0,1,2 and 6–12
	11-15 yrs	20micrograms	0 and 6–12^b^
Engerix B®	16 yrs. and over	40micrograms	0,1,2 and 6
Fendrix®	15 yrs. and over	20micrograms	0,1,2 and 6
^a^HBvaxPro Paediatric®	0-15 yrs.	5micrograms	0,1,2 and 6
HBvaxPro40®	16 yrs. and over	40micrograms	0, 1 and 6

^a^Although there is experience within the paediatric population of the use of this regime in children aged 0–15, this is strictly outside the product licence

^b^If high risk of acquiring infection with HBV during vaccination course, 3 dose or accelerated schedule as per manufacturer guidelines, should be used

#### Guideline 5.5 – BBV infection: identification and management of ‘responders’ to the immunisation programme

We recommend that patients should be regarded as an ‘adequate responder’ if the anti HBs antibody titre is >100mIU/ml 8 weeks after completing the immunisation schedule. (1C).

We recommend that responders to HBV immunisation should receive a further booster dose if the annual anti HBs titre is <100mIU/ml. (1B).

#### Guideline 5.6 – BBV infection: identification and management of ‘non-responders’ to the immunisation programme

We suggest that patients should be regarded as an inadequate-responder if the anti HBs antibody titre is < 100mIU/ml 8 weeks after completing the first complete immunisation schedule. (1C).

We would suggest the following strategies:

1. If the anti HBs Ab titre is between 10 IU/ml and 100 IU/ml we recommend administering a booster dose of the vaccine. (1C)
2. If the anti HBs titre is < 10 IU/ml we recommend repeating the entire vaccination course with the high concentration of the vaccine (or the appropriate dose for children age < 16 years). (1C) Follow up with an anti-HBs antibody titre test 4 to 6 weeks following the last injection to ensure it is greater than 10 m IU/l.
3. If after two full vaccination courses the Anti HBs titre remains <10mIU/ml we recommend that the patient is labeled as a non-responder to the vaccine, and therefore not immune to HBV.
4. A non-responder patient, who is therefore not immune to HBV, should be counselled about how to minimize risk of HBV exposure and the recommended actions needed to take in the advent of a potential Hepatitis B exposure (this is likely to include urgent receipt of Hepatitis B immunoglobulin). (1B)

#### Guideline 5.7 – BBV infection: management of patients prior to overseas travel or high risk exposure

We recommend that responders to the HBV vaccine should have the anti HBs titre checked prior to travel overseas or high risk exposure (1C), with a booster dose administered if the Anti HBs antibody titre is <100miU/ml. (1C).

### Immunisation of staff against Hepatitis B virus (guidelines 6.1–6.2)

#### Guideline 6.1 – BBV infection: immunisation of staff against hepatitis B

We recommend that staff members who have clinical contact with patients should be immunised against HBV and demonstrate that they are immune to, and are not infected with HBV. (1A). Staff members who have current infection with HBV require occupational health clearance and ongoing monitoring in order to perform clinical duties. They would not usually be employed to work clinically on a dialysis unit.

#### Guideline 6.2 – BBV infection: immunisation of staff against hepatitis B

We suggest that staff that are not immune to HBV and are not HBV infective should not dialyse patients who are HBV infective. (2B).

### Management of a new case of BBV infection in the Haemodialysis unit (guidelines 7.1–7.4)

#### Guideline 7.1 – BBV infection: management of a new case of Hepatitis B virus infection within the Haemodialysis unit

##### Guideline 7.1.1 – BBV infection: management of a new case of Hepatitis B virus infection within the Haemodialysis unit

**- Management of the incident case**

We recommend that when a new case of HBV infection is identified, the affected patient should be referred to HBV specialist for further evaluation and consideration of antiviral treatment.

##### Guideline 7.1.2 – BBV infection: management of a new case of Hepatitis B virus infection within the Haemodialysis unit - surveillance of prevalent HD population

We recommend that, whenever a previously unidentified case of HBV infection is identified, units should carry out enhanced HBV surveillance (as described in section 3.6) on all patients who are not adequately immune to HBV (anti HBs titre >100mIU/mL within the last six months) who have had a dialysis session in that unit since the index patient’s last negative test. (1B).

##### Guideline 7.1.3 – BBV infection: management of a new case of Hepatitis B virus infection within the Haemodialysis unit – immunisation of prevalent HD population

We recommend that, whenever a previously unidentified case of HBV infection is found, those patients who have anti-HB titre 10-100mIU/ml in the preceding six months, who have had a dialysis session in that unit since the index patient’s last negative test should also be given a booster dose of Hep B vaccine. Hepatitis B immunoglobulin (HBIG) should be considered for previous non-responders to Hepatitis B vaccine (anti-HBs <10mIU/ml) who may have been exposed in the previous 7 days (Table 2).

**Table 2:** Hepatitis B Immunoglobulin (HBIG) Dosage

Age Group	Dose
0-4 yrs	200 IU
5-9 yrs	300 IU
10 yrs and older	500 IU

#### Guideline 7.2 – BBV infection: management of a new case of Hepatitis C virus or HIV infection within the Haemodialysis unit

We recommend that, when a previously unidentified case of HCV is found, enhanced surveillance (as described in section 3.6) should be carried out in all patients who have had a dialysis session in that unit since the index patient’s last negative test. (1C).

#### Guideline 7.3 – BBV infection: management of any new BBV infection within the Haemodialysis unit

We recommend that, when a haemodialysis patient develops a new BBV infection, expert virological advice should be obtained to co-ordinate enhanced surveillance of at-risk dialysis patients and carers and to arrange treatment of affected individuals. (1C) An ‘outbreak group’ should be formed, which should include representatives from the infection prevention committee expert virologists in addition to staff from the haemodialysis service. This group will coordinate the response. A clearly documented enhanced screening process for contacts with identified staff responsibilities and regular review should be established.

#### Guideline 7.4 - BBV infection: review of practice within Haemodialysis units following any BBV infection

We recommend that, when there is a new case of a BBV infection within a haemodialysis unit, there should be a review of adherence to infection control procedures related to the management of BBV. There should be a review of cleaning and disinfection procedures.

## Summary of audit measures

**We recommend that the audits selected which prioritise specific areas of concern or challenge within Haemodialysis units.**

- **Audit Measure 1**: Adherence to the standard operating procedure for machine disinfection between haemodialysis sessions.
- **Audit Measure 2:** How frequent is contamination of external pressure monitor filters with blood or saline observed during haemodialysis sessions and what are the factors associated with contamination?
- **Audit Measure 3:** What proportion of prevalent dialysis patients are known to be immune to HBV (anti HBs > 10 mU/mL within the last year). Of the remainder, what proportion has a HBsAg test result from within the last 3 calendar months?
- **Audit Measure 4:** The proportion of incident patients starting regular hospital haemodialysis who have anti HBs antibody titre >100mIU/mL
- **Audit Measure 5**: The proportion of patients known to be infected with HBV who dialysed in a segregated area (using the DoH definition of ‘segregated’).
- **Audit Measure 6**: The proportion of patients who are expected to require RRT within two years who have initiated a HBV immunisation schedule.

## Rationale for clinical practice guidelines

### Prevention of BBV infection in the renal unit (guidelines 1.1–1.2)

#### Guideline 1.1- BBV prevention: infection control procedures

The single most important method of prevention of transmission of blood borne viruses is the rigorous application of universal infection control precautions. We recommend that infection control procedures must include hygienic precautions that effectively prevent the transfer of blood or fluids contaminated with blood between patients either directly or via contaminated equipment or surfaces (KDIGO Hepatitis C Guideline 3.1) (1A).

##### Rationale

The dialysis process facilitates transmission of BBV due to the considerable potential for exposure to blood. BBV can survive and remain potentially infective on surfaces of clinical equipment through splashes of blood that may not be visible to the naked eye [28, 29]. HCV ribonucleic acid (RNA) has been detected on the hands of nurses dialysing infected patients [30]. Whilst HBV deoxyribonucleic acid (DNA) and HCV RNA have been detected in the dialysate of patients known to have these infections, there is no evidence that the internal fluid pathways offer a viable route for transmission of BBV [31–33].

Units should adopt the highest standards of infection control as laid out in DoH regulations [3] and in the KDIGO guidelines for hepatitis C [34].

Universal precautions include:

- thorough hand washing after each patient contact and after contact with blood, body fluids or potentially blood-contaminated surfaces/ supplies.
- wearing of disposable gloves whenever caring for a patient or touching dialysis equipment; changing gloves and cleaning hands between patients every time.
- wearing of disposable plastic aprons/impermeable gowns when splashing with blood or body fluids may occur
- eye protection (visors, goggles, or safety spectacles) when blood, body fluids or flying contaminated debris/tissue might splash into the face
- staff covering any cuts or abrasions with waterproof plasters
- immediate and safe disposal of sharps into appropriate puncture-proof sharps bins
- not overfilling sharps containers (should not be filled to more than two-thirds capacity)
- never re-sheathing needles
- disposing of unused medications/ supplies (syringes/ swabs) taken to a dialysis station
- thorough inspection of dialysis machine including transducer protectors for contamination with blood
- thorough cleaning and disinfection of surfaces at the dialysis station
- adequate separation of clean supplies from contaminated materials and equipment

Implementing these precautions will require a plentiful supply of protective equipment, adequate hand washing facilities and adequate nursing and cleaning staff.

Particular attention should be paid to the layout of the dialysis unit; lighting, flow of ‘traffic’, heat and noise. Inadequacies in these areas can increase the risks of accidental exposure to blood. There should be adequate space between beds for staff to perform their clinical duties in a safe manner. Every effort should be made to avoid staff rushing clinical care, to minimise the opportunity for accidental transmission of blood from one patient to another. Recording machine numbers and position of machines for each dialysis session should be considered if possible, as this facilitates screening at risk population in the event of a new seroconversion. We also recommend units adopting strategies to minimise the movement of patients between dialysis machines - so that in the event of seroconversion the numbers exposed will be reduced. Studies in Italian [35] and Saudi Arabian [36] haemodialysis centres revealed a significant association between the incidence and prevalence of HCV and the level of staffing, suggesting that inadequate staffing plays a role in transmission.

Renal units should establish protocols for cleaning and disinfecting exposed surfaces and equipment in the dialysis unit with neutral detergent and hot water and thoroughly dried between patient treatments. For each chemical cleaning and disinfectant agent, units should follow the manufacturer’s instructions regarding appropriate dilution and contact time. Time between shifts should be sufficient to enable effective machine and surface decontamination. Any blood spillage should be immediately cleaned with a cloth soaked with an anti-microbial disinfectant or bleach. Shared equipment should be cleaned according to manufacturers’ instructions.

Implementation of these simple measures described above has been shown to be effective in preventing transmission when a patient has contracted BBV outside the renal unit and dialysed in the unit until BBV was detected by surveillance [37].

Infection control policies and practices should be audited on a monthly basis by infection prevention link nurses and infection prevention and control team in accordance with Saving Lives 2007 [38].

#### Guideline 1.2 – BBV prevention: use of parenteral medicines

We recommend that medicine vials should be discarded after single use and multi-use vials should be avoided. If medicine vials are used for more than one patient, we recommend these are divided into multiple doses and distributed from a central area (1B). Intravenous medication vials labelled for single use should not be punctured more than once, as the sterility of the product cannot be guaranteed once a needle has entered a vial labelled for single use [39].

##### Rationale

The use of multi-dose vials of medicines such as heparin, saline and lignocaine has been associated with avoidable outbreaks of HBV and HCV in dialysis units by facilitating needle contamination of the vial with an infected patient’s blood that is then transmitted to another patient via another needle [40–43]. Therefore the use of multi-dose vials is not recommended and instead use of sterile, single-use, disposable needles is.

recommended where possible [43]. If multi-vial compounds are used, medicines should be prepared and distributed from a central clean area removed from the patient treatment area [44, 45]. Infection control practice must be followed during preparation and administration of injected medications. We recommend a documented risk assessment and standard operating procedure is produced if multi use vials are regularly used.

**Audit Measure 1**: Adherence to the standard operating procedure for machine disinfection between haemodialysis sessions.

### Dialysis equipment and BBV infection (guidelines 2.1–2.5)

#### Guideline 2.1 – BBV infection: machine segregation for patients infected with HBV

We recommend that separate machines must be used for patients known to be infected with HBV (or at high risk of new HBV infection). A machine that has been used for patients infected with HBV can only be used again for non-infected patients after it has been decontaminated using a process recognised to be effective against HBV. Healthcare workers dialysing patients with known HBV infection should not dialyse patients without HBV infection at the same time (1A).

##### Rationale

HBV is highly infectious with significantly higher concentration of viral particles in an infected patient compared to HCV or HIV infected counterparts. A non-immune patient with an untreated percutaneous exposure to an infected source carries a risk of seroconversion of up to 30%; by contrast the risks of HCV and HIV are 1.8 and 0.3% respectively [46]. HBsAg positive patients who are also positive for hepatitis B e antigen have an extremely high viral load in their blood and are likely to have appreciable levels of HBV in body fluids containing serum or blood [47]. HBV is relatively stable in the environment and has been shown to remain viable for at least 7 days on environmental surfaces (including clamps, scissors, dialysis machine control buttons and door handles) at room temperature [48] in the absence of visible blood and still contain high viral titres. There is strong epidemiological evidence that segregation of HBV infected dialysis patients reduces HBV transmission among dialysis patients [49].

For these reasons patients with chronic HBV infection (HBsAg positive or evidence of circulating viral DNA) should be dialysed using dedicated dialysis machines and staff, in a segregated area or rooms [47], with no sharing of instruments, medications and supplies between patients, regardless of serological status [48]. Segregated area refers to an area with physical barriers such as walls or screens ensuring there is no possibility of traffic between infected and clean areas. Healthcare workers dialysing patients with known HBV infection should not dialyse patients without HBV infection at the same time. Environmental surfaces including dialysis chair/ bed, external surface of HD machine, clamps etc. must be thoroughly decontaminated using a process recognised to be effective against HBV after each use.

Standard disinfection of machines between patients does not eliminate the risk of transmission of HBV [50]. A machine that has been used for patients infected with HBV can be used again for non-infected patients only after it has been thoroughly decontaminated using a process recognised to be effective against HBV. A local protocol for decontamination should be drawn up, taking into account the manufacturer’s instructions, the design of the machine and the use of double transducer protectors. The pressure transducer ports should be decontaminated after each use unless double transducer protectors are routinely used. If the machine does not automatically disinfect the Hansen connectors, they should be disinfected manually (e.g. by immersion in bleach for 10 min). If the machine housing is known to have points that are vulnerable to blood seepage, these should be checked and disinfected.

#### Guideline 2.2 – BBV infection: precautions for patients with HCV/HIV

We recommend that dedicated machines are not required for patients infected with HCV and HIV, provided cleaning and disinfection procedures are strictly adhered to between patients [34, 51] (KDIGO Hepatitis C guidelines) (1D).

There is no evidence to support the use of dedicated dialysis machines for patients infected with HCV [52]. Transmission of HCV through internal pathways of modern single-pass dialysis machines has not been demonstrated (KDIGO Hepatitis C Guidelines 3.1). Transmission would require the virion to cross the intact dialyser membrane, migrate from the drain tubing to the fresh dialysate circuit and pass through the dialyzer membrane of a second patient, although the virus cannot cross the intact membrane. Even in the event of a blood leak, transmission would require HCV to reach fresh dialysate used for a subsequent patient and enter the blood compartment of that patient through back-filtration across the dialyser membrane. This very low theoretical risk of HCV transmission via the haemodialysis circuit could be eliminated altogether by using double transducer protectors for patients who are HCV positive [33]. In isolated cases of HCV transmission a role for the dialysis circuit could not be excluded, but the environmental surfaces are more likely to have contributed to transmission [53].We therefore do not recommend the use of dedicated dialysis machines for individuals infected with HCV.

We do not suggest isolation of HCV-infected patients during HD is strictly necessary to prevent direct or indirect transmission of HCV. However, given the low prevalence of HCV in dialysis patients, it would be reasonable for individual units to consider isolating patients who are HCV RNA positive, if facilities are available. This should not be at the expense of rigorous universal infection control procedures.

Given the low likelihood of patient-to-patient and/or patient-to-staff transmission of HIV, dedicated machines for HIV-positive patients undergoing haemodialysis is not recommended [54, 55]. Strict adherence to universal infection control procedures can avoid the risk of HIV transmission in haemodialysis patients, although the evidence is limited [56, 57].

#### Guideline 2.3 – BBV infection: utilisation of external transducer protectors

We suggest that external transducer protectors on the blood circuit pressure monitoring lines should be inspected by healthcare personnel during and after each dialysis session. If there is evidence of breach by blood or saline then the machine should be taken out of service and machine components that may have come in contact with blood should be replaced or decontaminated by qualified personnel according to a protocol that incorporates the manufacturers’ instructions. (2C).

**Audit Measure 2:** How frequent is contamination of external pressure monitor filters with blood or saline observed during haemodialysis sessions and what are the factors associated with contamination?

##### Rationale

Transducers serve an important role in monitoring the pressures within the arterial and venous circuits. Transducer filter protectors act as a barrier between the blood in the tubing and the internal transducer in the machine. Haemodialysis machines usually have both external (typically supplied with the blood tubing set) and internal protectors, with the internal protector serving as a backup in case the external transducer protector fails.

Moisture can damage the pressure transducer. Therefore leaking of these filters (‘breaches’) can occur especially if wetting with saline or blood has compromised the integrity of the filter. Failure to use an external protector or to replace the protector when it becomes contaminated (i.e., wetted with saline or blood) can result in contamination of the internal transducer protector, which in turn could allow transmission of blood borne pathogens. There are reports of leaks associated with these protective systems [58–60], as well as reports of nosocomial transmission of BBV that could implicate contamination of the dialysis machine due to undetected failures of the external filter [61, 62].

Wet external transducer protectors must be changed immediately, and the machine side of the protector should be inspected for contamination or wetting. If a fluid breakthrough is found on the removed transducer protector, the machine’s internal transducer protector must be inspected by a qualified technician, for safety, quality, and infection control purposes. In the unlikely event that the internal filter ruptures, the machine must be taken out of service and decontaminated according to a local protocol that incorporates the manufacturer’s instructions.

There are several measures that can reduce the risk of breach of these filters:

- monitoring the blood levels in the arterial and venous drip chambers during the haemodialysis session with adjustment as required to prevent overfilling;
- stopping the blood pump before resetting arterial or venous pressure alarms;
- clamping the venous and arterial monitoring bloodlines before removing them from the machine at the end of the dialysis session.

Some units now routinely add a second external transducer protector filter in series with the one already fitted to the pressure monitoring line which reduces the need for technical interventions that take the machine out of service.

#### Guideline 2.4 – BBV infection: disinfection process for dialysis equipment

We recommend that the dialysis machine should be cleaned between patients according to a local protocol that incorporates the manufacturer’s instructions. (1C).

##### Rationale

Cleaning of dialysis machines between patients is a key component of the efforts to minimise the risk of BBV transmission in the renal unit. Dialysis units should establish protocols for cleaning and disinfecting surfaces and equipment in the dialysis unit, including, where appropriate, careful mechanical cleaning before any disinfection process. For each chemical cleaning and disinfectant agent the manufacturer’s instructions regarding appropriate dilution and contact time should be followed. The internal fluid pathways should also be cleaned according to the manufacturer’s instructions.

HBV DNA and HCV RNA have been detected in dialysate of patients known to have these infections [32, 63] although it is doubtful if a contaminated dialysis fluid circuit has ever been the direct source of nosocomial infection.

The KDIGO Hepatitis C guidelines [34] are included in Table 3, to summarise hygienic precautions for dialysis machines to minimise the risk of BBV transmission.

**Table 3:** KDIGO Hepatitis C guideline summary of hygienic precautions for dialysis machines. Reproduced from reference [34]

Hygienic precautions for dialysis machines	
**Definitions** The ‘transducer protector’ is a filter (normally a hydrophobic 0.2-mm filter) that is fitted between the pressure monitoring line of the extracorporeal circuit and the pressure monitoring port of the dialysis machine. The filter allows air to pass freely to the pressure transducer that gives the reading displayed by the machine, but it resists the passage of fluid. This protects the patient from microbiologic contamination (as the pressure monitoring system is not disinfected) and the machine from ingress of blood or dialysate. An external transducer protector is normally fitted to each pressure monitoring line in the blood circuit. A back-up filter is located inside the machine. Changing the internal filter is a technical job. A ‘single-pass machine’ is a machine that pumps the dialysate through the dialyser and then to waste. In general, such machines do not allow fluid to flow between the drain pathway and the fresh pathway except during disinfection. ‘Recirculating’ machines produce batches of fluid that can be passed through the dialyser several times.	
**Transducer protectors** External transducer protectors should be fitted to the pressure lines of the extracorporeal circuit. Before commencing dialysis, staff should ensure that the connection between the transducer protectors and the pressure-monitoring ports is tight as leaks can lead to wetting of the filter. Transducer protectors should be replaced if the filter becomes wet, as the pressure reading may be affected. Using a syringe to clear the flooded line may damage the filter and increase the possibility of blood passing into the dialysis machine so it is essential to fit a new transducer protector to the monitoring line if this procedure has to be used. If wetting of the filter occurs after the patient has been connected, the line should be inspected carefully to see if any blood has passed through the filter. If any fluid is visible on the machine side, the machine should be taken out of service at the end of the session so that the internal filter can be changed and the housing disinfected.	
**External cleaning** After each session, the exterior of the dialysis machine should be cleaned with a low-level disinfectant if not visibly contaminated. If a blood spillage has occurred, the exterior should be disinfected with a commercially available tuberculocidal germicide or a solution containing at least 500 p.p.m. hypochlorite (a 1:100 dilution of 5% household bleach) if this is not detrimental to the surface of dialysis machines. Advice on suitable disinfectants, and the concentration and contact time required, should be provided by the manufacturer. If blood or fluid is thought to have seeped into inaccessible parts of the dialysis machine (for example, between modules, behind blood pump), the machine should be taken out of service until it can be dismantled and disinfected.	
**Disinfection of the internal fluid pathways** It is not necessary for the internal pathways of a single-pass dialysis machines to be disinfected between patients, unless a blood leak has occurred, in which case both the internal fluid pathways and the dialysate-to-dialyser (Hansen) connectors should be disinfected before the next patient. If machines are not subjected to an internal disinfection procedure, staff should ensure that sufficient time is available between patients for the external surfaces to be disinfected. Machines with recirculating dialysate should always be put through an appropriate disinfection procedure between patients.	

### BBV surveillance in dialysis patients (guidelines 3.1–3.6)

#### Guideline 3.1 – BBV infection: virology status of patients starting Haemodialysis

We recommend that all patients starting haemodialysis (including patients with acute kidney injury) or returning to haemodialysis after another modality of renal replacement therapy should be known to be HBsAg negative before having dialysis on the main dialysis unit. (1A).

We recommend HCV screening all patients starting haemodialysis or returning to haemodialysis after another modality of renal replacement therapy. We recommend patients with no identified risk factors for acquiring HCV may be screened by serological methods followed by reflex nucleic acid test (NAT) if serology is reactive. Patients with ongoing risk factors should be screened by NAT (KDIGO Hepatitis C guideline 1.2.2) (1A).

We recommend that HIV screening should be undertaken in all patients starting haemodialysis (1C).

#### Guideline 3.2 – BBV infection: management of patients starting Haemodialysis with unknown virology status

We recommend that patients who require haemodialysis before the result of the HBsAg test is known should be dialysed in an area that is segregated from the main dialysis unit and the machine should not be used for another patient until the result is known to be negative or the machine has been thoroughly decontaminated (see 2.1) (1A).

The DoH report 2002 defined segregation between infected and clean areas in a renal unit as being ‘functionally complete with no possibility of traffic between the two’ and suggested there be a physical barrier such as walls or screens between these infected and clean areas.

#### Guideline 3.3 – BBV infection: surveillance for HBV/HCV/HIV in prevalent Haemodialysis population

We recommend that patients on regular hospital haemodialysis who are immune to hepatitis B infection (annual anti HBs antibody titre > 100 mIU/ml; see section 5 below), need to be tested for HBsAg every 6 months. Non-responders and those with inadequate response should be tested at least every 3 months (1C).

We recognise that there are challenges in implementing a testing regime where different timings can be employed depending on antibody titres. For this reason units may prefer to routinely test for HBsAg every 3 months.

We recommend that patients on regular hospital haemodialysis, without any identified ongoing risk factors for HCV acquisition, should be tested for HCV antibody at least every 3-6 months (1C). A patient specific screening plan utilising NAT testing should be initiated for patients with on-going HCV acquisition risks.

We recommend that antibody surveillance testing for HIV is not necessary for patients on regular hospital haemodialysis unless the patient is at high risk (see Table 4) (1C).

**Table 4:** Patients at high risk for new BBV infection (adapted from National Institute on Drug Abuse website [64]

Risk factors for new BBV infection	
• Injection drug use	
• Male to male sexual contact	
• Commercial sex workers	
• Sexual contact with partners who inject illicit drugs or have BBV infection	
• Infected with other BBV	
• Recent kidney transplant from a donor known to be infected with BBV	
• Recent receipt of health care in intermediate/ high risk countries	

#### Guideline 3.4 – BBV infection: management of patients who do not consent for BBV testing

We suggest that patients who do not consent to BBV surveillance as described above should have dialysis in a segregated area unless they are known to be HBV immune in the previous 6 months. If patients who are known to be HBV immune in the previous 6 months do not consent to BBV surveillance then they should be managed in the same way as patients with HCV infection (see section 4) (2C).

**Audit Measure 3:** What proportion of prevalent dialysis patients are known to be immune to HBV (anti HBs > 10 mU/mL within the last year). Of the remainder, what proportion has a HBsAg test result from within the last 3 calendar months?

**Audit Measure 4:** The proportion of incident patients starting regular hospital haemodialysis who have anti HBs antibody titre >10mIU/mL.

##### Rationale (for 3.1–3.4)

BBV infections are asymptomatic in the majority of individuals and therefore a surveillance system is required to detect new BBV infection and implement measures to limit the opportunity for nosocomial spread [3, 65]. The frequency of surveillance testing should be determined in part by patient specific risk factors, the local prevalence and incidence of infection. The UK is a low prevalence country for BBV infection in patients with.

Established renal failure and so surveillance can be less frequent than in higher risk countries [34]. Surveillance needs to be enhanced if the patient’s overall risk is high or if the individual patient experiences an event that increases the risk. Our previous guidelines have recommended patients on regular hospital haemodialysis who are immune to hepatitis B infection (annual anti HBs antibody titre > 100 mIU/ml) only need to be tested for HBsAg once a year. However, antibody titres can fall over time, leading some patients to become unprotected. In a US study [66], 8% of chronic haemodialysis patients became unprotected due to a fall in antibody titres over a 12 month period. For this reason, we recommend testing this group of patients on a 6 monthly basis.

For those who are not immune to HBV infection, we recommended HBsAg testing at least every 3 months for normal risk patients. Testing for HBsAg is sufficient for the diagnosis of HBV infection in the majority of dialysis patients. However occult HBV infection (the presence of HBV DNA detectable by real time PCR in the absence of detectable HbsAg) has been reported in 1.3–3.8% of chronic haemodialysis patients [34, 67], although the risk in UK is likely to be considerably lower. There are reports of transmission of HBV infection from patients with occult HBV infection though, to date, not in association with haemodialysis [67–69]. NAT may be indicated in such isolated cases.

Patients who have antibodies to the hepatitis B core antibody (Anti HBc) are at increased risk of viral reactivation compared to those who are core antibody negative. This patient group should be screened at least 3 monthly. The risk of viral reactivation is increased during periods of immunosuppression. We would recommend vaccination of this cohort - and use of prophylactic antiviral therapy in situations where the risk of reactivation is enhanced.

HBsAg testing should not be performed within 2 weeks of receipt of a Hepatitis B vaccine as the assay may detect the vaccine and cause concern that there is current infection [70]. If testing and vaccination are undertaken at similar time points the serum sample should be drawn before the vaccine is administered.

Our previous guidelines [38] and KDIGO guidelines recommend 6 monthly testing for HCV antibody using a 3rd generation assay [34]. HCV antibody tests are unable to distinguish between resolved HCV infection and current HCV infection. In addition HCV antibodies may not be detectable for several months after HCV infection [71]. In these patients HCV RNA positive result would indicate current infection. Patients who are HCV antibody-positive and HCV RNA-negative have resolved infection but remain at risk for re-infection if exposed [72]. Detection of HCV viraemia relies on NAT technologies. Therefore patients who are HCV antibody positive and HCV RNA negative (i.e. those with resolved infection), should undergo screening for HCV reinfection every three to six months using NAT.

The probability of acquiring HIV infection in UK dialysis units is very low and therefore does not justify regular surveillance for otherwise low risk patients. However, unless there is a robust system of routinely questioning patients to assess for risks of new BBV infection, there is a potential to miss new cases of BBV. Therefore many units routinely screen for HIV antibody on a 6–12 monthly basis. Similarly, in an attempt to reduce complexity with BBV surveillance, many units in the UK routinely screen for HBsAg and HCV on a 3 monthly basis in all patients and this approach is perfectly acceptable.

#### Guideline 3.5 – BBV infection: management of patients returning from dialysis outside UK

We recommend that patients planning to dialyse outside the UK should have a risk assessment prior to travel for potential exposure to BBV abroad. Where exposure is considered likely, enhanced surveillance testing for BBV should be planned and instituted and patients should have dialysis in a segregated area as detailed below (1B).

##### Rationale

Good practice guidelines for renal dialysis and transplant units by DoH [71] provides guidance on classifying countries at low, medium or high risk of BBV exposure for patients dialysing away from base (see Appendix 1). Prior to travel units should review the immunisation status of the patient and administer booster vaccinations if needed.

Individual units may wish to undertake a risk assessment of the planned DAFB unit (accepting that the assessment of risk is subjective), counsel patients regarding the potential risks of BBV infection and the plans for segregation and surveillance on return. The level of risk for BBV exposure will depend on the prevalence of BBV in the country visited [73], infection control policies in the DAFB unit and lifestyle activities of individual patients.

On return from DAFB, patients should be risk assessed for potential exposure to BBV whilst abroad. Examples of questions to be included in this risk assessment on return are highlighted in Appendix 2.

Depending on the risk of BBV exposure we recommend the following level of surveillance: 
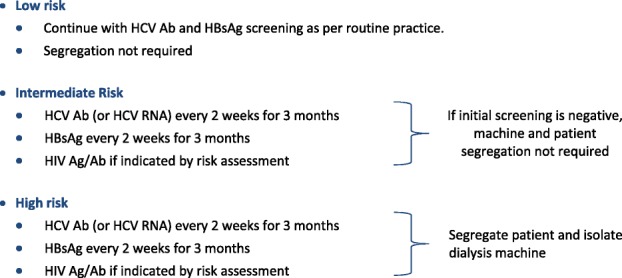


Our previous guidelines have suggested that enhanced surveillance for HBV is not required if immune with HBsAb level > 100 mIU/mL in the last 12 months. However, antibody titres can fall over time, leading some patients to become unprotected. In view of this and in an attempt to reduce the level of complexity in the guidelines, which can lead to errors if misinterpreted, we have recommended same level of surveillance irrespective of HBsAb levels.

#### Guideline 3.6 – BBV infection: procedures for enhanced surveillance of high risk patients

We recommend that patients at high risk for new BBV infection (see Table 4) should have enhanced surveillance as described in 3.5 (1B).

We recommend that testing for HBsAg and HCV RNA should be performed in haemodialysis patients with unexplained abnormal serum aminotransferase concentrations (KDIGO Hepatitis C guideline 1.2.3) (1B).

We recommend that if a new BBV infection is identified in a haemodialysis unit, testing for viral RNA or DNA should be performed in all patients who may have been exposed (see section 7) (KDIGO Hepatitis C guideline 1.2.4) (1B).

##### Rationale

It makes sense to adopt the improved assays to detect acute BBV infection in patients at increased risk for BBV infection. Detection of HCV RNA by PCR techniques has the advantage of significantly shortening the window period from infection to positive result compared to serological methods. HCV RNA may be detectable within 1–2 weeks of infection, whereas antibodies may take up to a year to be detectable in immunosuppressed individuals [34]. Furthermore, some patients with HCV infection do not develop detectable antibody. HCV core antigen testing may be available in certain laboratories and has a similar window period to RNA testing, however concerns about limits of sensitivity mean it is not a recommended as a complete replacement for RNA testing currently [74, 75].

Newly infected patients with HBV and particularly HCV infection may have an increase in ALT levels prior to antibody conversion. Therefore baseline, followed by monthly monitoring of serum ALT in susceptible patients has been recommended to enable early detection of new HCV infection in patients receiving haemodialysis [74]. Unexplained elevated ALT levels should prompt additional evaluation for HBV/HCV infection. Because few haemodialysis patients newly infected with HCV report symptoms or have symptoms documented in their dialysis medical records, ALT levels are also often used retrospectively to define the likely exposure period for patients who acquired infection, thus narrowing the focus of a HCV case investigation to the most likely exposure and source.

Acquisition of a new BBV infection should prompt immediate evaluation of all other patients in the same facility to identify additional cases. The virology status of all at risk patients should be reviewed and all uninfected patients should be tested for BBV. The frequency of repeat screening should be increased for a limited time. For example, monthly testing for 3 months, followed by testing again in 3 months, and then resumption of screening every 6 months if no additional infections are identified [55, 76]. Identification of BBV transmission within a dialysis facility should prompt re-evaluation of infection control practices and need for corrective action.

We recommended that all new cases of BBV infection identified on the dialysis unit should be referred to the hepatology/ virology team for consideration for treatment, to reduce individual and population risk.

### Segregation of patients infected or at risk of infection with BBV (guidelines 4.1–4.2)

#### Guideline 4.1 – BBV infection: isolation of patients known to be infected with Hepatitis B virus (HBV)

We recommend that patients infected with HBV must be dialysed in an area that is segregated from the main dialysis unit. (1A).

We recommend that healthcare workers performing dialysis on patients infected with HBV infection should not dialyse patients without HBV infection at the same time. (1C). If this is not possible then they must wear PPE and ensure thorough decontamination before moving from one patient to the other.

**Audit Measure 5**: The proportion of patients known to be infected with HBV, are dialysed in a segregated area (using the DoH definition of ‘segregated’).

##### Rationale

The DoH report 2002 defined segregation between infected and clean areas in a renal unit as being “functionally complete with no possibility of traffic between the two” and suggested there be a physical barrier such as walls or screens between these infected and clean areas.

There is ample evidence that suggests ‘horizontal’ (patients not sharing a machine) and ‘vertical’ (patients sharing a machine) transmission of HBV occurs when patients infected with HBV are dialysed beside uninfected patients [3, 77]. The risk of HBV transmission has been shown to be reduced if patients infected with HBV are dialysed in an area that is segregated from the “clean” area of the dialysis unit [78–80]. Transmission has been reported in situations where health workers care for infected and non-infected patients on the same haemodialysis shift. This also applies for HBV infected patients undergoing invasive procedures (such as central venous catheter insertion) on the dialysis unit. Such procedures should take place in a segregated area. BBV can survive and remain infective on surfaces of clinical equipment, even where blood splashes are not visible to the naked eye. Any unused equipment (syringes, swabs, spare catheters) taken into the room where the procedure has occurred should be disposed of [3, 80].

#### Guideline 4.2 – BBV infection: management of patients infected with Hepatitis C virus (HCV) or HIV

We recommend that patients with HCV or HIV do not need to be dialysed in a segregated area, providing infection control and universal precautions can be properly adhered to (1C). (KDIGO Hepatitis C guideline 3.1).

##### Rationale

The risk of nosocomial transmission is much lower for HCV and HIV than HBV. In a study from Italy HCV RNA was detected on the outer surface of the inlet-outlet connector of a dialysis machine used for HCV non-infected patients but there was no evidence of any patients becoming infected [29]. Data from the Dialysis Outcomes and Practice Patterns (DOPPS) study indicated that HCV seroconversion was equivalent whether patients with HCV were segregated or not segregated for haemodialysis [81]. Similarly a prospective multi-centre Belgian study showed that re-enforcement of universal precautions without segregation was sufficient to reduce the incidence of HCV infection from 1.41 to 0.8% and this is supported by other observational studies [20, 24, 82, 83]. In a large prospective multicentre study in the USA there were no cases of HIV infection in the subsequent year in centres where universal precautions were in place and where other patients with HIV were being dialysed [23].

The most important factor implicated in HCV transmission between patients treated in the same dialysis unit is cross-contamination from supplies and surfaces as a result of failure to follow infection control procedures [20].Our previous guidelines from 2008 [84], CDC [54], recent KDIGO Hepatitis C guidelines (3.1.3) [34] and European Best Practice Work Group [85] have not recommended routine isolation of patients infected with HCV in a segregated area to prevent HCV transmission.

Studies that have reported reduction in HCV transmission following isolation, have been observational studies with poor quality evidence [86, 87], often comparing results to historical controls, leading to lack of clarity as to whether the improvements were a result of the isolation policy or concurrent increased awareness and reinforcement of universal infection control policies during the studies [7, 89, 90].

These observations are re-assuring but the numerous reports of HCV and HIV transmission in dialysis units [8, 10, 18, 19, 91–96] emphasise the importance of local monitoring of the implementation of infection control procedures outlined in sections 2 and 3. There is evidence from areas with a high prevalence of HCV infection that segregation is associated with reduced nosocomial infection, both from a randomised control trial in Iran [32] and observational studies [19, 36, 88, 96, 97] though isolation should not be seen as a substitute for strict contamination control procedures.

For a low BBV prevalence country like the UK it seems reasonable to propose segregation facilities are prioritised for patients with HBV infection but are also used for patients with HCV and HIV infection if there are concerns about the implementation of contamination control procedures. It is for this reason that paediatric patients with any BBV are often dialysed in a segregated area.

The same principles should apply to patients with BBV who are admitted for in-patient care in the renal unit. Every effort must be made to ensure that these measures do not compromise the care of the patient being segregated.

### Immunisation of patients against Hepatitis B virus (guidelines 5.1–5.7)

#### Guideline 5.1 – BBV infection: indications for immunisation of patients against hepatitis B virus (HBV)

We recommend that all patients who require renal replacement therapy (RRT) [dialysis or transplantation] for CKD should be assessed for current or past infection with Hepatitis B and offered vaccination against HBV if indicated. (1A).

##### Rationale

The introduction of HBV immunisation was associated with a reduction in the incidence of HBV infection in dialysis units [78].

A randomised controlled trial of immunisation suggested a reduction in HBV infection [98] and a case controlled study demonstrated a 70% reduction in HBV infection in patients who had received HBV immunisation compared with those who had not [99].

Despite the lower probability of HBV infection in peritoneal dialysis patients compared with HD patients [100] patients planning to have peritoneal dialysis should also be immunised as there is a sufficiently high probability that they will require haemodialysis at some point.

Pre-emptive renal transplantation has become the treatment of choice for end stage kidney disease. Candidates for such a method of RRT should be vaccinated against HBV in the pre-transplant period. This is because seroconversion rates in renal allograft recipients on immunosuppression is much lower (36%) even when vaccinated with an enhanced scheme (4 × 40 μg of the recombinant vaccine), whilst recipients vaccinated before transplantation developed an adequate anti-HBs titre in 86% of cases [101].

Passive immunisation with HBV immunoglobulin was previously shown to be effective in reducing the incidence of HBV infection in patients and staff in dialysis units [102] but this has now been superseded by active immunisation. HBV immunoglobulin is now exclusively available for post exposure protection in a limited number of scenarios [103].

#### Guideline 5.2 – BBV infection: timing of initiating immunisation schedule against HBV

We recommend that patients who are likely to require RRT should be offered immunisation prior to the development of Stage V CKD [or 2 years before they are likely to need renal replacement therapy]. (1A) A kidney failure risk calculator could be used to this prediction.

##### Rationale

The proportion of patients achieving adequate anti HBs antibody titres after immunisation is lower in patients with CKD than in the general population [104–106] and is lower in advanced CKD compared with milder stages of CKD. [101, 102, 107–112].

**Audit Measure 6**: The proportion of patients who are expected to require RRT within two years who have initiated a HBV immunisation schedule.

#### Guideline 5.3 – BBV infection: identification of patients for whom immunisation against HBV is not indicated

Hepatitis B vaccine is not indicated in patients who have current (Hepatitis B surface antigen (HBsAg) positive or HBV DNA positive) or confirmed past HBV infection. Presence of the anti HBc antibody in isolation should not be taken as confirmation of previous HBV infection. Patients identified to be core antibody positive who are at risk of reactivation of HBV (particularly immunosuppression) may need to be vaccinated and the case should be discussed with a local virologist. (2B).

##### Rationale

Although there is no documented harm associated with the administration of the HBV vaccine to patients with natural immunity, it is recommended that anti-HBc and anti-HBs antibodies should be checked prior to immunisation. Patients who have a positive anti HBs antibody and who have a detectable anti HBc usually have natural immunity to HBV and therefore may not need vaccination. However, detection of Hepatitis B core antibody should not be used in isolation to determine immunity or previous infection and these patients may still require vaccination.

The need for pre-immunisation screening for anti HBc to avoid unnecessary immunisation should be guided by the likelihood that an individual has been exposed to HBV or previous vaccine as a study in the USA suggests that pre-immunisation screening is cost-effective only in populations in which the prevalence of HBV infection exceeds 30% [113].

Hepatitis B core antibody detected reports can arise from many scenarios. (Table 5 - interpretation of HBV results prior to vaccination).

- Recent receipt of blood products (core antibody is passively acquired and is a frequent finding in patients who have received blood, plasma, IVIg or similar in the last few weeks, testing a serum sample predating the blood products is required to determine patient status), (HBV vaccination will be required)
- Occult infection: HBV DNA will be detected and Anti HBs antibody levels are usually low (HBV vaccination not required)
- False positive: discussion with local virology team to determine if referral to reference lab can be helpful (HBV vaccination required)

**Table 5:** Interpretation of HBV results prior to vaccination (1B)

HBsAg	anti-HBs titre	Anti-HBc	Interpretation
–	–	–	**Not Immune** Has not been infected, but still at risk for possible future infection. **VACCINATE**
–	+	+	**Immune** Surface antibodies present due to previous infection, and now recovered. **VACCINE NOT NEEDED**
–	+	–	**Immune** Has already been vaccinated. Level of immunity will depend on titre. REFER to medical staff if NO prior history of vaccination **VACCINE MAY / MAY NOT BE NEEDED**
+	–	+	**Hepatitis B Infection** Hepatitis B virus is present. REFER to medical staff **VACCINE NOT NEEDED**
–	–	+	**Unclear** likely natural immunity - vaccination may be indicated particularly in immunocompromised patients

Although patients are routinely considered as having HBV transmission in the past and not infectious to others, there is an increasing evidence that these persons may replicate or may start to replicate under special circumstances (immunosuppression, cachexia) [111]. Any patient with confirmed past HBV infection who is going to be significantly immunosuppressed is at risk of reactivation and a pre-emptive management plan should be made with a Hepatitis B specialist.

#### Guideline 5.4 – BBV infection: immunisation schedule for vaccination against Hepatitis B virus

We recommend that the initial HBV immunisation schedule should involve high doses, frequent doses or both of the available preparations.

We recommend that the vaccines are administered intramuscularly as per their licensed route (deltoid muscle) but, if sufficient expertise exists, the intradermal route may more effective [114]. (1A) (Table 1).

The DOH has now developed a model patient group direction for use of HBV vaccines in advanced renal failure - https://www.gov.uk/government/publications/hepatitis-b-vaccine-for-renal-patients-patient-group-direction-template

We recognise that there is a fine balance to be had with frequent hospital attendances versus pragmatic vaccination schedules. Although schedules indicated provide immunity as rapidly as possible, some flexibility around scheduling is possible with, for instance, vaccinations given every 3 months to tie in with appointments. The vital element is to ensure that there is a gap of at least 4 weeks between first and second vaccine. Extension of the vaccine schedule prolongs time to protection, but longer intervals between the doses do improve immune response. Patients should be tested 4–8 weeks after their primary immunisation course for evidence of response, and annually thereafter, with booster doses, as required.

##### Rationale

There are several reports of increased success of immunisation if higher individual doses of vaccine are used, a greater number of doses are given, and if the intradermal route is used [115–120].

Most studies have shown that a 4 dose double dose schedule over 6 months is superior to the conventional 3 dose immunisation schedule [121, 122]. This is also logistically easier than identifying non-responders to the 3 dose schedule and administering a booster dose.

There is some evidence that HBV vaccine with the adjuvant ASO4 (Fendrix) is more immunogenic than Engerix B [123].

There has been recent interest in adding immunostimulants to improve the success of HBV immunisation in patients with CKD [29–32] but it is too early to make a firm recommendation and reports have, in some cases, had conflicting conclusions [124, 125].

The World Health Organisation recommended universal childhood vaccination against HBV in 1992 and by 2003, 79% of member states had implemented this. The UK adopted this approach in August 2017. Countries that have implemented this have seen exceptional falls in their childhood prevalence rates of Hepatitis B. Cost-benefit analyses have strongly supported the introduction of universal vaccination against HBV to newborns, outside the UK, as part of a vaccination programme [126, 127]. Results of children’s vaccination, which were evaluated in the six-year outcome of the programme, showed neither new cases of HBsAg de novo nor seroconversion to anti-HBc positivity [128].

#### Guideline 5.5 – BBV infection: identification and management of ‘responders’ to the immunisation programme

We recommend that patients should be regarded as an ‘adequate responder’ if the anti HBs antibody titre is >100mIU/ml 8 weeks after completing the immunisation schedule. (1C).

We recommend that responders to HBV immunisation should receive a further booster dose if the annual anti HBs titre is <100mIU/ml. (1B).

##### Rationale

Response should be assessed by measuring plasma anti HBs antibody 8 weeks after completion of the immunisation schedule. There is on-going debate about what constitutes a response to immunisation.

Conventionally >100mIU/ml was regarded as conferring immunity but there is evidence that even patients who have a lower peak response (10-100mIU/ml) will not become chronic carriers of HBV [65, 129].

The significance of this titre was illustrated in a five-year follow-up study of 773 homosexual men vaccinated in 1980; most severe infections occurred among those who never achieved a titre > 9.9mIU/ml. The risk of late infection in those with an initial titre of > 9.9mIU/mL increased markedly when antibody levels decreased below 10mIU/mL, but only 1 of 34 of these late infections resulted in viraemia and liver inflammation [129].

In a series of haemodialysis patients, with anti HBs antibody titres >10mIU/ml who received transplants from HBsAg positive donors, 67% seroconverted to anti-HBc positivity - suggesting that such an anti-HBs titre does not always protect against HBV infection in HD patients [111].

It is worth being aware of the possibility of HBV surface mutants that can cause HBV infection in patients with apparently adequate anti HBs titres and seem to occur in endemic regions with large HBV vaccination programs (vaccine escape mutants) [130, 131].

More than half of haemodialysis patients who respond to immunisation do not maintain detectable antibody [132]. In one of the early randomised controlled studies of immunisation there were 4 cases of hepatitis B infection in dialysis patients who had an apparent response to immunisation in whom the antibody levels had waned, suggesting a strategy of antibody surveillance and booster doses may be worthwhile [98].

In one small Italian study the monitoring of antibody titres and the administration of additional doses enabled maintenance of protective HBV antibody levels in 96% of patients 4 years after initial immunisation [132].

Retrospective reviews from the 1990s convincingly demonstrate a higher response in non-dialysis compared to dialysis patients (80% compared to 50% in one analysis) [110, 112]. In 2003 Da Rosa et al. prospectively demonstrated that GFR was an independent positive predictive variable of seroconversion in response to the vaccination [108].

Previous guidelines have also recommended annual testing of patients who have ever achieved a HBV titre >10mIU/mL with administration of a booster dose of vaccine if titre < 100mIU/ml but we acknowledge that the frequency of surveillance and the titre to trigger a booster dose is debatable.

#### Guideline 5.6 – BBV infection: identification and management of ‘non-responders’ to the immunisation programme

We suggest that patients should be regarded as an inadequate-responder if the anti HBs antibody titre is < 100mIU/ml 8 weeks after completing the first complete immunisation schedule. (1C).

We would suggest the following strategies:

1. If the anti HBs Ab titre is between 10 IU/ml and 100 IU/ml we recommend administering a booster dose of the vaccine. (1C)
2. If the anti HBs titre is <10 IU/ml we recommend repeating the entire vaccination course with the high concentration of the vaccine (or the appropriate dose for children age < 16 years). (1C) Follow up with an anti-HBs antibody titre test 4 to 6 weeks following the last injection to ensure it is greater than 10 m IU/l.
3. If after two full vaccination courses the anti HBs titre remains <10mIU/ml we recommend that the patient is labeled as a non-responder to the vaccine, and therefore not immune to HBV.
4. A non-responder patient, who is therefore not immune to HBV, should be counselled about how to minimize risk of HBV exposure and the recommended actions needed to take in the advent of a potential Hepatitis B exposure (this is likely to include urgent receipt of Hepatitis B immunoglobulin). (1B)

##### Rationale

We recommend that non-responders to HBV should receive no further immunisation - the likelihood of benefit is low, compared to the cost burden. However there is some evidence that non-responders to a 4 dose 40 μg schedule might subsequently respond to a large dose given intra-dermally [132]. In high risk groups (potential transplant recipients, individuals intending to dialyse away from base in a high risk area) this should be considered. Anecdotal evidence suggests that those more likely to respond to second courses include non-smokers, low BMI, age < 40, or high levels of immunosuppressive drugs at the time of the first vaccination.

A non-responder patient, who is therefore not immune to HBV, should be counselled about how to minimize risk of HBV exposure and recommended actions needed to take in the advent of a potential Hepatitis B exposure (this is likely to include urgent receipt of Hepatitis B immunoglobulin). (1B).

They should also be advised about the risks of overseas travel and dialysis away from base. Patients who are not immune to HBV who dialyse in units where the prevalence of risk of HBV is higher should undergo a period of enhanced surveillance on their return to the UK, and be dialysed on their own machine, ideally in a segregated area. (1C).

#### Guideline 5.7 – BBV infection: management of patients prior to overseas travel or high risk exposure

We recommend that responders to the HBV vaccine should have the anti HBs titre checked prior to travel overseas or high risk exposure (1C), with a booster dose administered if the Anti HBs antibody titre is <100miU/ml. (1C).

##### Rationale

Haemodialysis patients who mount a good response to vaccine appear unable to maintain high antibody levels. Fleming et al. showed that 57% of haemodialysis patients who mounted a good response had lost detectable anti-HBs within 6 months of immunisation [133], and therefore will need a booster dose of the vaccine to maintain their immunity.

### Immunisation of staff against Hepatitis B virus (guidelines 6–1 – 6.2)

#### Guideline 6.1 – BBV infection: immunisation of staff against Hepatitis B virus

We recommend that staff members who have clinical contact with patients should be immunised against HBV and demonstrate that they are immune to, and are not infected with HBV. (1A). Staff members who have current infection with HBV require occupational health clearance and ongoing monitoring in order to perform clinical duties. They would not usually be employed to work clinically on a dialysis unit.

#### Guideline 6.2 – BBV infection: immunisation of staff against Hepatitis B virus

We suggest that staff that are not immune to HBV and are not HBV infective should not dialyse patients who are HBV infective. (2B).

##### Rationale

Several reports of outbreaks of HBV and HCV infection in dialysis units have included patient to staff and staff to patient transmission. Staff members are at much lower risk of acquiring HIV or HCV infection than HBV infection [134–136]. It is important, therefore to have a mechanism in place to minimise this risk.

Hepatitis B immunisation of dialysis unit staff members has been shown to be effective in reducing the incidence of HBV infection in these staff members [106, 137]. Staff who are in contact with clinical equipment that might be infected with HBV should also be offered HBV immunisation (e.g. dialysis technicians).

Staff members are at very low risk of acquiring HIV or HCV from dialysis patients.

### Management of a new case of BBV infection in the Haemodialysis unit (guidelines 7.1–7.4)

#### Guideline 7.1 – BBV infection: management of a new case of Hepatitis B virus infection within the Haemodialysis unit

##### Guideline 7.1.1 – BBV infection: management of a new case of Hepatitis B virus infection within the Haemodialysis unit

We recommend that when a new case of HBV infection is identified, the affected patient should be referred to HBV specialist for further evaluation and consideration of antiviral treatment.

Antiviral therapy against HBV is effective in reducing the viral load to undetectable levels and as a result reducing both the infectivity of the patient and the potential for long term sequelae of HBV.

##### Guideline 7.1.2 – BBV infection: management of a new case of Hepatitis B virus infection within the Haemodialysis unit - surveillance of prevalent Haemodialysis population

We recommend that, whenever a previously unidentified case of HBV infection is identified, units should carry out enhanced HBV surveillance (as described in section 3.6) on all patients who are not adequately immune to HBV (anti HBs titre >100mIU/mL within the six months) who have had a dialysis session in that unit since the index patient’s last negative test. (1B).

##### Guideline 7.1.3 – BBV infection: management of a new case of Hepatitis B virus infection within the Haemodialysis unit – immunisation of prevalent Haemodialysis population

We recommend that, whenever a previously unidentified case of HBV infection is found, those patients who have anti-HB titre 10-100mIU/ml in the preceding 6 months, who have had a dialysis session in that unit since the index patient’s last negative test should also be given a booster dose of Hep B vaccine. Hepatitis B immunoglobulin (HBIG) should be considered for previous non-responders to Hepatitis B vaccine (anti-HBs <10mIU/ml) who may have been exposed in the previous 7 days. (1B) (Table 2).

#### Guideline 7.2 – BBV infection: management of a new case of Hepatitis C virus or HIV infection within the Haemodialysis unit

We recommend that, when a previously unidentified case of HCV is found, enhanced surveillance (as described in section 3.6) should be carried out in all patients who have had a dialysis session in that unit since the index patient’s last negative test. (1C).

#### Guideline 7.3 – BBV infection: management of any new BBV infection within the Haemodialysis unit

We recommend that, when a haemodialysis patient develops a new BBV infection, expert virological advice should be obtained to co-ordinate enhanced surveillance of at-risk dialysis patients and carers and to arrange treatment of affected individuals. (1C) An ‘outbreak group’ should be formed, which should include representatives from the infection prevention committee expert virologists in addition to staff from the haemodialysis service. This group will coordinate the response. A clearly documented enhanced screening process for contacts with identified staff responsibilities and regular review should be established.

We recommended that all new cases of BBV infection identified on the dialysis unit should be referred to the hepatology/ virology team for consideration for treatment, to reduce individual and population risk. All patients should be counselled regarding the implications of having a blood borne virus and the risk of infectivity. Success rates for treatment of HBV and HCV have increased over the last 10 years and all patients should be considered for antiviral therapy.

Following successful treatment there will need to be on-going surveillance for reinfection. The risk of HCV reinfection is between 1 and 8% in those undertaking high risk behaviours. [138] We would recommend that even after remission has been confirmed (HCV PCR negative) then precautions described within the guidelines should be observed.

For patients who successfully undergo treatment for HBV and who become HepBsAg negative we would recommend that, given the risk of viral reactivation, enhanced precautions are maintained and the patients should dialyse on a dedicated machine.

#### Guideline 7.4 - BBV infection: review of practice within Haemodialysis units following any BBV infection

We recommend that, when there is a new case of a BBV infection within a haemodialysis unit, there should be a review of adherence to infection control procedures related to the management of BBV. There should be a review of cleaning and disinfection procedures.

##### Rationale

Whenever a new case of blood borne virus infection is identified in the renal unit there is a risk that other patients may be incubating the same infection. For this reason it is necessary to perform enhanced surveillance of all at-risk patients [69–71, 74, 75, 139, 140]. The screening should be coordinated and regularly reviewed by a senior member of the unit staff to ensure all patient contacts are adequately followed up.

In addition the risk of spread of HBV within the renal unit may be reduced by passive immunisation of non-responders to HBV vaccine using HBIG and by the administration of a booster dose of Hepatitis vaccine to all patients who had borderline HBsAb B titres in the preceding 12 months.

The assistance of the local virology and infection prevention and control services in co-ordinating surveillance and prevention measures is invaluable. The virology service should be requested to supervise the overall management of the new BBV infection(s) until no further cases are detected.

When Hepatitis B and C outbreaks in health care settings have been reviewed, the majority of outbreaks relate to incomplete adherence to infection control procedures - both standard - and those specific to haemodialysis. Cases due to machine contamination were rare (1 in 16) as were cases due to use of contaminated blood products [25, 27, 141].

## Data Availability

Not applicable.

## Appendix 1

### Guidance on classifying risk of BBV exposure for patients dialysing away from base

**Table:**

**Low risk countries:** UK, Europe, US, Canada, Australia, New Zealand and Japan	
**High risk countries:** Indian subcontinent, parts of Africa	
**Intermediate risk countries:** Rest of the world including South East Asia, South America, Middle East	

**Adapted from:** Department of Health. Good Practice Guidelines for Renal Dialysis/Transplantation Units, Prevention and Control of Blood-Borne Virus Infection - Addendum, Guidelines for Dialysis Away From Base (DAFB). 2010.

https://assets.publishing.service.gov.uk/government/uploads/system/uploads/attachment_data/file/382208/guidelines_dialysis_away_from_base.pdf

## Appendix 2

### Examples of questions to be included in local risk assessment on return from DAFB

**Table:**

• While abroad did you have any blood transfusions?	
• While abroad did you have any surgery or dental treatment?	
• While abroad were you ill, requiring hospital admission?	
• Were any needles, dialysis lines or dialysers shared between you or any other patients?	
• Do you undertake any high risk sexual activity?	
• Do you inject any intravenous drugs into yourself?	

**Adapted from:** Department of Health. Good Practice Guidelines for Renal Dialysis/Transplantation Units, Prevention and Control of Blood-Borne Virus Infection - Addendum, Guidelines for Dialysis Away From Base (DAFB). 2010.

https://assets.publishing.service.gov.uk/government/uploads/system/uploads/attachment_data/file/382208/guidelines_dialysis_away_from_base.pdf

## Abbreviations

ALT: Alanine amino transferase
BBV: Blood borne virus
CDC: Centers for Disease Control and Prevention
CKD: Chronic Kidney Disease
DAFB: Dialysis away from base
DOPPS: Dialysis Outcome and Practice Patterns Study
HAART: Highly Active Anti-Retroviral Therapy
HBIG: Hepatitis B Immunoglobulin
HBsAb: Hepatitis B surface antibody
HBsAg: Hepatitis B surface antigen
HBV: Hepatitis B Virus
HCV: Hepatitis C Virus
HD: Haemodialysis
HepBcAb: Hepatitis B core antibody
HIV: Human immunodeficiency virus
IU: International Units
KDIGO: Kidney Disease: Improving Global Outcomes
NAT: Nucleic Acid Test (ing)
PCR: Polymerase Chain Reaction
RNA: Ribonucleic Acid
